# The effect of diabetes on the risk of endometrial Cancer: an updated a systematic review and meta-analysis

**DOI:** 10.1186/s12885-019-5748-4

**Published:** 2019-05-31

**Authors:** Lotfolah Saed, Fatemeh Varse, Hamid Reza Baradaran, Yousef Moradi, Sorour Khateri, Emilie Friberg, Zaher Khazaei, Saeedeh Gharahjeh, Shahrzad Tehrani, Amir-Babak Sioofy-Khojine, Zahra Najmi

**Affiliations:** 1 Department of Endocrinology, Faculty of Medicine, Kurdistan University of Medical Sciences, Sanandaj, Iran; 2 Department of Epidemiology, School of Public Health, Iran University of Medical Sciences, Tehran, Iran; 3 Endocrine Research Center, Institute of Endocrinology and Metabolism, Iran University of Medical Sciences, Tehran, Iran; 4 Ageing Clinical & Experimental Research Team, Institute of Applied Health Sciences, University of Aberdeen, Aberdeen, UK; 5 Student Research Committee, Kurdistan University of Medical Sciences, Sanandaj, Iran; 6 Division of Insurance Medicine, Department of Clinical Neuroscience, Karolina Institute, Stockholm, Sweden; 7 Department of Public Health, Iranian Research Center on Healthy Aging, Sabzevar University of Medical Sciences, Sabzevar, Iran; 8 Department of Infertility, Infertility Fellowship, Tehran University of Medical Sciences, Tehran, Iran; 9 Department of Gynecology and Obstetrics, Qom University of Medical Sciences, Qom, Iran; 10 Faculty of Medicine and Health Technology, Tampere University, Arvo Ylpön katu 34, FI-33520 Tampere, Finland; 11 Obstetrics and Gynecology, Fellowship of Minimally Invasive Gynecology, Zanjan University of Medical Sciences, Zanjan, Iran

**Keywords:** Diabetes, Endometrial Cancer, Risk, Meta-analysis

## Abstract

**Background:**

Previous studies conducted on the association between diabetes and the risk of endometrial cancer have reported controversial results that have raised a variety of questions about the association between diabetes and the incidence of this cancer. Thus, the aim of this systematic review and meta-analysis was to more precisely estimate the effect of diabetes on the risk of endometrial cancer incidence.

**Methods:**

All original articles were searched in international databases, including Medline (PubMed), Web of sciences, Scopus, EMBASE, and CINHAL. Search was done from January 1990 to January 2018 without language limitations. Also, logarithm and standard error logarithm relative risk (RR) were used for meta-analysis.

**Results:**

A total of 22 cohort and case-control studies were included in this meta-analysis, of which 14 showed statistically significant associations between diabetes and risk of endometrial cancer. Diabetes was associated with increased risk of endometrial cancer (RR = 1.72, 95% CI 1.48–2.01). The summary of RR for all 9 cohort studies was 1.56 (95% CI 1.21–2.01), and it was 1.85 (95% CI 1.53–2.23) for 13 case control studies. The summary of RR in hospital-based studies was higher than other studies. Thirteen of the primary studies-controlled BMI as a confounding variable, and the combined risk of their results was 1.62 (95% CI 1.34–1.97).

**Conclusions:**

Diabetes seems to increases the risk of endometrial cancer in women, and this finding can be useful in developing endometrial cancer prevention plans for women having diabetes.

## Background

A recent study conducted by Lortet-Tieulent, J and colleague show that endometrial cancer is the sixth most commonly occurring cancer in women and the 15th most commonly occurring cancer overall. There were over 380,000 new cases in 2018 [1]. Also, about 142,000 women are diagnosed with endometrial cancer annually worldwide, and about 42,000 women lose their life due to endometrial cancer. The usual curve of endometrial cancer indicates that most cases are diagnosed after menopause, and the highest incidence rate is around the seventh decade of life [2]. The disease is more than 10 times common in North America and Europe than in less developed countries [3]. The incidence and the mortality rate of endometrial cancer increased during 2006 and 2010 [4]. Estrogens, both internal and external, play an important role in increasing endometrial cancer [5]. Several studies have shown that the risk of endometrial cancer increases with older age, early menstruation, late menopause, obesity, family history of endometrial cancer (especially among close relatives), exposure to radiation, infertility (especially due to polycystic ovarian syndrome), and long-term use of estrogens for hormone therapy [4–7]. Estrogens, both internal and external, play an important role in increasing endometrial cancer. Multiple studies have claimed a positive association between diabetes and incidence of endometrial cancer with several biological mechanisms [8]. However, a previous systematic review and meta-analysis was performing by Friberg and colleges [8] but growing several publications afterwards and also considering new variables in adjusted models, we felt to design an updated systematic review and meta-analysis in order to show any posible relationship between diabetes and endometrial cancer.

## Methods

This systematic review was performed according to the Meta-Analyses of Observational Studies in Epidemiology (MOOSE) and Strengthening the Reporting of Observationally Studies in Epidemiology (STROBE) guidelines for reviews of analytical observational studies (case-control and cohort) [2, 9, 10].

### Search strategy

All original published articles were searched in international databases, including Medline (PubMed), Web of sciences, Scopus, EMBASE, and CINHAL. Search was done from January 1990 to January 2018 without language limitations. The keywords were Diabetes, Diabetes Mellitus (type 1 and 2), Insulin Dependent, IDDM, NIDDM, Noninsulin Dependent, Endometrial Stromal Tumors, Endometrial Neoplasms, and Endometrial. The selected studies were limited to observational studies on humans.

**Fig. 1:**
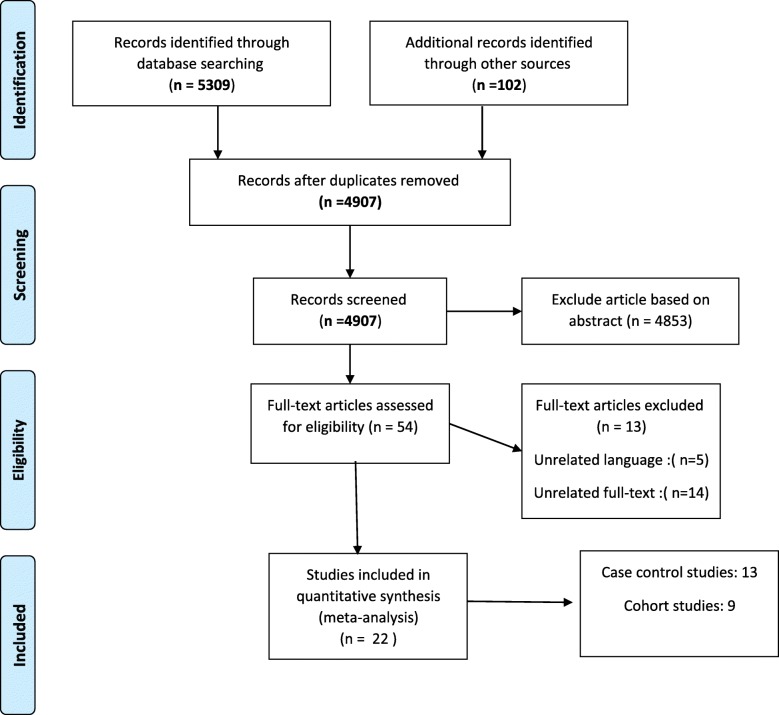
Flow Diagram of the Literature Searches and Study Selection

The primary search results were reviewed, and some of the articles were eliminated after reviewing their title and an abstract. Inclusion and exclusion criteria were set by 2 researchers separately (YM, FV) (Fig. 1).

### Eligibility criteria

A published study had to meet the following inclusion criteria:

(1) original article, (2) case-control or cohort study, (3) human population, (4) diabetes and patients with diabetes as the main independent variable, and (5) endometrial cancer as the dependent variable. Case reports, reviews, animal studies, and case control or cohort studies with crude estimates about the effect of diabetes on the risk of endometrial cancer were removed from the tabulation. The authors resolved all disputes during the collection, compilation, and analysis of data.

### Data extraction

Two researchers evaluated all included articles independently. They assessed the disagreement, if any, and in case an agreement was not reached, a third author (LS) evaluated the study. Two independent matched reviewers extracted the data according to a uniform Excel sheet. Then, a structured checklist was used to extract the following information: (1) author, (2) year of publication, (3) type of study, (4) country, (5) study population, (6) age of women, (7) sample size, (8) type of diabetes, (9) measurement, and (10) adjusted variables of association.

### Statistical analysis

In the meta-analysis, 3 measures of association were used: (1) odds ratio (case-control and population-based case-control studies), (2) relative risk (cohort and population cohort studies), and (3) hazard ratio (cohort and population-based cohort studies). As the frequency of endometrial cancer was relatively low, the odds ratio in the case-control and population based case-control studies and the risk ratio in the cohort and population-based cohort studies yielded similar estimates of relative risk (RR) [11].

Logarithm and standard error logarithm relative risk (RR) were used for the meta-analysis. DerSimonian and Laird method was used to compute the pooled estimate of relative risk (RR) with confidence interval (CI 95%) using random models [12]. Because the test for heterogeneity was statistically significant in some analyses, the random effects models were used to estimate RR. In this study, w Cochran’s Q test and I2 statistic were used to evaluate statistical heterogeneity between studies [13]. In addition, a meta-regression and subgroup analysis was performed to assess the source of heterogeneity between studies. Moreover, publication bias was assessed by funnel plot and Egger and Begg’s test [14, 15]. Statistical analysis was performed using STATA 14.0 (Stata Corp, College Station, TX, USA), and statistical significance was set at *p* < 0.05.

## Results

### Study characteristics

A total of 22 studies were included in this meta-analysis (Fig. 1), of which 9 were cohort and population cohort studies [4–6, 16–21] (Table 1) and 13 were case-control and population case-control studies [22–34] (Table 2). Also, 12 studies were conducted in the USA [4, 5, 16, 19, 20, 22, 27, 29–32, 34], 4 in Sweden [17, 18, 21, 33], 2 in Italy [25, 26], 1 in Canada [23], 1 in Norway [6], 1 in Mexico [28], and 1 in Japan [24]. The case-control and population case-control studies (*n* = 13) comprised 22,392 controls and 7698 endometrial cancer cases.

**Table 1:** The Main Characteristics of Cohort and Population-based Cohort Studies on Diabetes and Endometrial Cancer Risk

Authors	Year	Type of study	Country	Study population	Age	Sample size	Type of diabetes	Measurement of association	Controlled variables
Al Hilli. M, et al. [16]	2015	Cohort	USA	database for the records of all patients who underwent primary surgical intervention for EC, from January 1, 1999, through December 31, 2008.	All age	1303	Diabetes	HR: 1.01; % 95 CI (0.72,1.42)	Age, BMI
Friberg. E, et al. [17]	2007	Cohort	Sweden	Exposed group: 1628 women with self-reported DM or DM from national inpatient register Comparison group: 35145 women without self-reported DM or DM from national inpatient register	50–83	36,773	Diabetes	RR: 1.94; % 95 CI(1.23,3.08)	Age, BMI, total physical activity
Anderson. K, et al.[5]	2001	Cohort	USA	Exposed group: 1325 women with self-reported DM Comparison group: 23150 women without self-reported DM	55–69	24,475	Diabetes	RR: 1.43; % 95 CI(0.98,2.09)	Age, BMI, BMI2, WHR, ovulatory span, gravidity, PMH, menstrual irregularities, hypertension
Lindemann. K, et al. [6]	2008	Cohort	Norway	Norwegian women during 15.7 years of follow-up.	All age	36,761	Diabetes	RR: 3.84 (% 95 CI: 1.92,5.11)	Age
Folsom. A, et al. [20]	2004	Cohort	USA	Exposed group: 42 women with self-reported DM and an endometrial cancer diagnosis Comparison group: 373 women with self-reported DM and an endometrial cancer diagnosis	55–69	415	Diabetes	RR: 2.38 (% 95 CI: 1.05,5.37)	Age, extent of endometrial cancer at diagnosis
Luo. J, et al. [4]	2014	Cohort	USA	Women’s Health Initiative	50–79	88,107	Diabetes	HR: 1.16 (% 95 CI: 0.90,1.48)	Age, BMI
Terry. P, et al. [21]	1999	Cohort	Sweden	Exposed group: 142 women with self-reported DM Comparison group: 10012 women without self-reported DM	42–81	10,154	Diabetes	RR: 1.60 (% 95 CI: 0.20,11.30)	Age, physical activity, weight, parity
Coughlin. S, et al. [19]	2004	Cohort	USA	Exposed group: 33 women with self-reported DM Comparison group: 448 women without self-reported DM	> 30	481	Diabetes	RR: 1.33 (% 95 CI: 0.92,1.90)	Age, race, education, BMI, smoking, alcohol, red meat, citrus fruit and juice, vegetables, physical activity, PMH, parity, age at menarche, age at first live birth, menopausal status, OC
esLambe. M, et al. [18]	2011	Cohort	Sweden	individuals that took part in routine health checkups and primary care patients referred for laboratory testing	All age	230,737	Diabetes	HR: 1.46(% 95 CI 1.09,1.96	Age

**Table 2:** The Main Characteristics of Case-Control and Population Case-Control Studies on Diabetes and Endometrial Cancer Risk

Authors	Year	Country	Control subjects (selection methods)	Age	Sample size	Type of diabetes	Measurement of association	Controlled variables
Weiderpass. E, et al. [33]	2000	Sweden	Control women were randomly selected from a continuously updated population register that includes all residents.	50–74	Case(709)	Diabetes	OR: 1.7 (% 95 CI: 1.2,2.3)	Age, age at menarche, parity, age at last birth, age at menopause, smoking, OC, PMH, BMI
					Control(3368)			
					T(4077)			
Shoff. SM, et al. [30]	1998	USA	Community controls were selected randomly from lists	40–79	Case(723)	Diabetes	OR: 1.10 (% 95 CI: 0.66,1.86)	Age, BMI, smoking, PMH, parity, education
					Control(2291)			
					T(3014)			
Lucenteforte. E, et al. [25]	2007	Italy	Controls women admitted to the same network of hospitals	18–79	Case(777)	Diabetes	OR: 2.0 (% 95 CI: 1.4,2.9)	Age, year of interview, study center, education, parity, menopausal status, OC and HRT use
					Control(1550)			
					T(2327)			
Friedenreich. CM, et al. [23]	2011	Canada	Controls selected from the Alberta Cancer Registry	30–79	Case(515)	Diabetes	OR: 1.31(95% CI: 1.03,1.67)	Age, parity, education, age at menarche, hormone therapy, age at menopause, history of Type 2 diabetes, hormone contraception, oral and non-oral hormone use, history of angina, history of stroke, history of thrombosis, smoking and alcohol consumption
					Control(962)			
					T(1447)			
Saltzman. BS, et al. [29]	2007	USA	Controls selected from Women’s Contraceptive and Reproductive Experiences (CARE) breast cancer study	45–74	Case(1303)	Diabetes	OR: 1.7(% 95 (CI: 1.2, 2.3)	Country, age, reference year, body mass index, and menopausal hormone use
					Control(1779)			
					T(3082)			
Parazzini. F, et al. [26]	1999	Italy	Controls selected from same network of hospitals where cases had been identified.	28–74	Case(752)	Diabetes	OR: 3.1 (% 95 CI: 2.3,4.2)	Age, calendar year, education, BMI, parity, OC, PMH, age at menopause, hypertension, smoking
					Control(2606)			
					T(3358)			
Wartko. PD, et al. [32]	2017	USA	Control were randomly selected from all other women with delivery records from 1987 to 2013.	All age	Case(593)	Diabetes	OR: 1.80 (% 95 CI: 1.22,2.65)	Race/ethnicity, year of delivery, maternal age at delivery, and body mass index
					Control(5743)			
					T(6336)			
Soliman. PT, et al. [31]	2006	USA	Controls patient samples were obtained through a low-risk cancer screening program	All age	Case(117)	Diabetes	OR: 1.87 (%95, CI: 0.77,4.54)	Lower serum adiponectin level, age, BMI, and hypertension
					Control(238)			
					T(355)			
Rubin. GL, et al.[27]	1990	USA	population controls, matched for place of residence and age	20–54	Case(196)	Diabetes	OR: 1.80 (%95, CI: 0.90,3.60)	Age
					Control(986)			
					T(1182)			
Brinton. L A, et al. [22]	1992	USA	Population controls random digit dialing for younger controls and health care financing administration for older controls, older controls were matched on age, race and zip code	20–74	Case(405)	Diabetes	OR: 1.95 (%95, CI: 1.10,3.60)	Age, education, number of births, weight, OC, PMH
					Control(279)			
					T(684)			
Inoue. M, et al. [24]	1994	Japan	hospital control who underwent hysterectomy due to benign gynecological tumors, matched on year of admittance to hospital and age	22–79	Case(143)	Diabetes	OR: 7.75 (%95, CI: 1.52,40.0)	Age, parity, cancer history, hypertension, obesity
					Control(143)			
					T(286)			
Weiss. J M, et al. [34]	2006	USA	Population that matched on age	45–75	Case(1281)	Diabetes	OR: 1.58 (%95, CI: 1.20,2.07)	Age, PMH, BMI, county, referent year, tumors aggressiveness
					Control(1779)			
					T(3060)			
Salazar. M E, et al.[28]	2000	Mexico	Hospital, from primary health center i.e. outpatient, matched on age	NA	Case(85)	Diabetes	OR: 3.60 (%95, CI: 1.70,7.40)	Age, an ovulatory index, smoking, physical activity, menopausal status, hypertension, BMI
					Control(668)			
					T(753)			

**Fig. 2:**
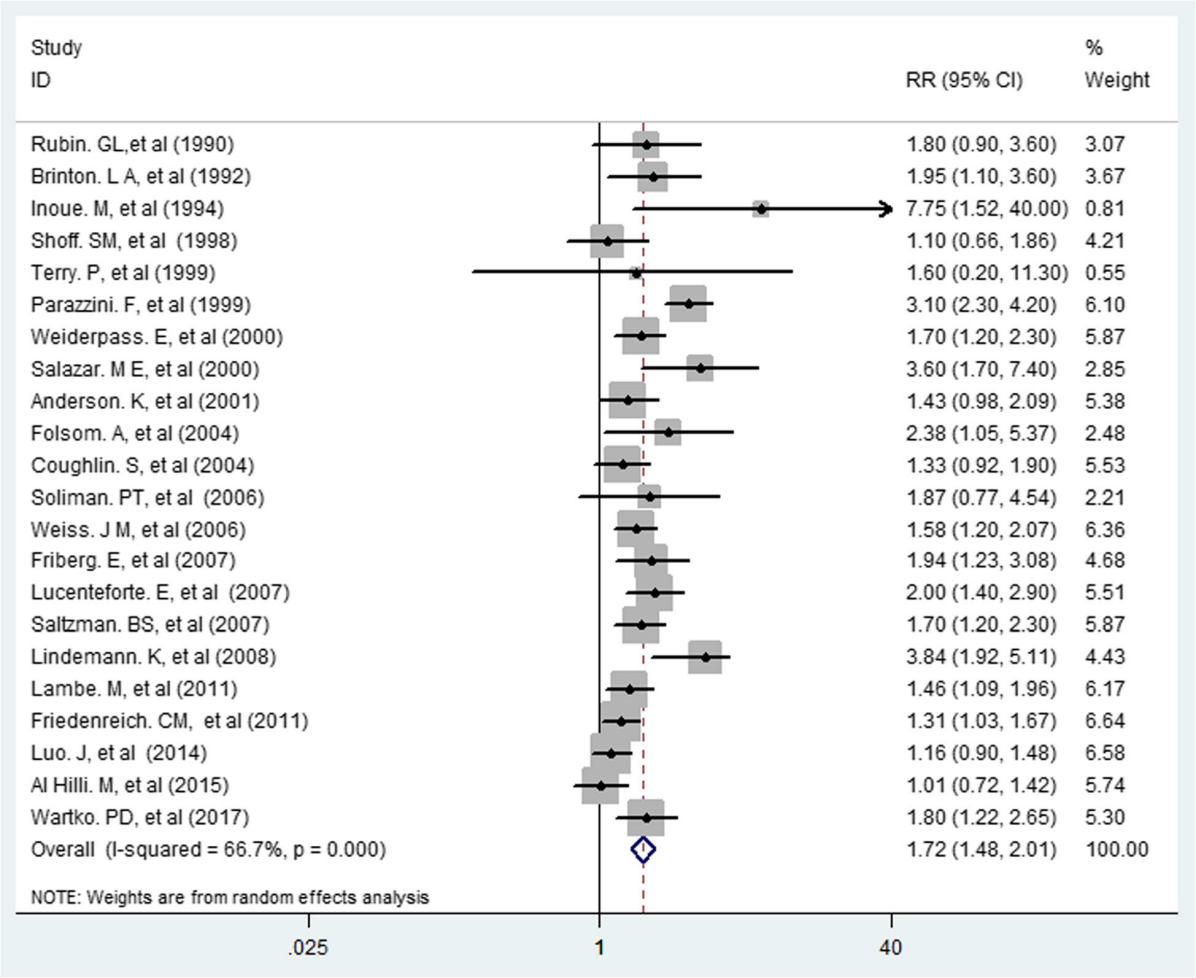
Association between Diabetes and Risk of Endometrial Cancer

**Fig. 3:**
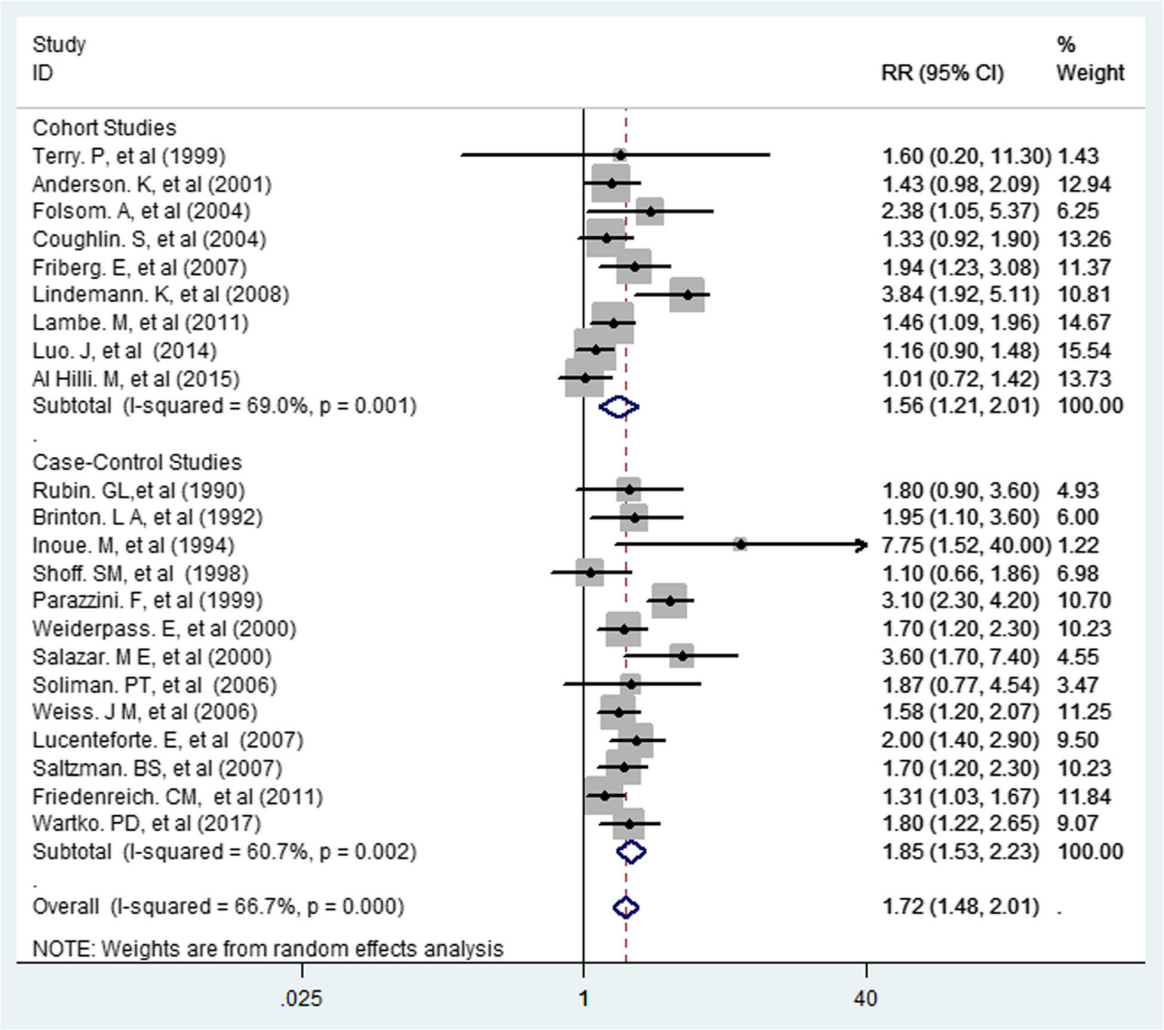
Association between Diabetes and Risk of Endometrial Cancer by Type of Study

The overall and individual results of 22 cohort and case-control studies are shown in Fig. 2. Of the 22 studies, 14 showed statistically significant associations between diabetes and risk of endometrial cancer. Occurrence of diabetes had an association with increased risk of endometrial cancer (RR = 1.72, 95% CI 1.48–2.01) (Figs. 2 and 3). The results demonstrated heterogeneity of the studies (I2 = 66.7%; *P* < 0.0001). However, no evidence of publication bias was found based on the results of the Egger’s test (Egger’s test: t = 1.90, *P* = 0.072, 95% CI: − 0.04-0.91).

### Subgroup analysis

The subgroup analysis was conducted based on the study design, and variables adjustment (Table 3). Individual study results and the overall summary results for 8 cohorts and 7 population-based, 2 hospital-based, and 5 case-control studies investigating the effect of diabetes on the risk of endometrial cancer in women are shown in Table 3. The results indicated that the summary of RR for all the 8 cohort studies combined was 1.52 (95% CI 1.16–2.00), and heterogeneity among these studies was significant (Q = 3.03, I^2^ = 70.7%; *P* = 0.001). The summary of RR for all the 7 population-based case–control studies was 1.55 (95% CI 1.37–1.75), however, heterogeneity among these studies was not significant (Q = 6.88, I^2^ = 0.0%; *P* = 0.461). In addition, the summary of RR for all the 5 case-control studies was 2.31 (95% CI 1.81–2.96), but heterogeneity was not significant (Q = 6.69, I^2^ = 22.7%; *P* = 0.270). Also, the summary of RR was higher in hospital-based studies than in other studies [RR = 4.10 (CI 95% 2.09–8.01), heterogeneity was Q = 4.12, I^2^ = 0.0%, *P* = 0.402]. According to the results in Table 3, the summary of RR in hospital-based studies was higher than in other studies. Also, 13 of the primary studies-controlled BMI as a confounding variable, and the combined risk of their results was 1.62 (95% CI 1.34–1.97, test for heterogeneity: Q = 4.14, I2 = 71.0%, *P* = 0.0001). However, 4 of the primary studies-controlled weight as a confounding variable, and the combined risk of their results was 2.45 (95% CI 1.14–5.26, test for heterogeneity: Q = 2.53, I2 = 21.0%, *P* = 0.021).

**Table 3:** Summary Relative Risk (RR) Estimates [95% confidence intervals (CIs)] for Case–Control and Cohort Studies Conducted on the Association Between Diabetes and Endometrial Cancer Incidence by Study Design, Continent, and Age

Subgroup	Number of studies	Summery Relative Risk (95% CI)	Between studies			Between subgroups	
			I^2^	P _heterogeneity_	Q	Q	P _heterogeneity_
Study design							
Cohort	8	1.52 (1.16–2.00)	70.7%	0.001	3.03	5.79	0.001^a^
Case-Control	5	2.31 (1.81–2.96)	22.7%	0.270	6.69		
Population-based	7	1.55 (1.37–1.75)	0.0%	0.461	6.88	4.95	0.034^b^
Hospital-based	2	4.10 (2.09–8.01)	0.0%	0.402	4.12		
Adjustment	3	1.88 (1.48–2.38)	81.9%	0.004	5.21	8.78	0.045
Age	13	1.62 (1.34–1.97)	71.0%	0.0001	4.14		
BMI	3	2.45 (1.14–5.26)	21.0%	0.021	2.53		
Weight	4	1.89 (1.22–2.94)	50.6%	0.108	6.08		
Physical Activity							

Largely diabetes mellitus

All statistical tests were 2-sided

^a^Test for heterogeneity between case-control and cohort studies

^b^Test for heterogeneity between population-based and hospital-based case-control studies

Also, the summary of RR of primary studies, whose results were adjusted based on BMI showed a less value compared to summary of RR of primary studies, whose results were adjusted based on weight control (1.62; 95% CI 1.34–1.97 Vs 2.45; 95% CI 1.14–5.26). Physical activity was adjusted in 4 primary studies, and the summary of RR based on controlling this variable was 1.89 (95% CI 1.22–2.94, test for heterogeneity Q = 6.08, I2 = 50.6%, *P* = 0.108) (Table 3).

## Discussion

The results of this meta-analysis showed that women with diabetes had a 72% increased risk of endometrial cancer compared to those without diabetes as supports the previous meta-analysis conducted by E. Friberg et al. (31) in 2007. Also, other studies have shown that diabetes increased the risk of endometrial cancer, which is in line with the results of the present study [5, 6, 16, 23, 26, 32, 35].

Based on subgroup analysis, the risk of endometrial cancer in case-control studies was higher than in cohort studies, and a higher risk was observed in hospital-based studies compared to population-based studies. [36–38].

Since the results of case-control and hospital-based studies are more prone to be affected by confounders therefore the calculated risk might be over-estimated. [39–41].

In our meta-analysis, heterogeneity was 66.7% for overall risk, which was reduced by subgroup analysis based on type of study, so the heterogeneity for each group for RR in cohort studies, case-control studies, population-based studies, and hospital-based studies were 70.7, 22.7, 0, and 0%, respectively. Furthermore, in this study, it was found that the levels of heterogeneity in physical activity, weight, and BMI had decreased remarkably. It can be concluded from the analysis that the causes of heterogeneity in determining the overall risk of endometrial cancer in women with diabetes in the present meta-analysis were type of study, adjusted co-variables and geographical area (Fig. 2).

Obesity, which is one of the most important factors in diabetes, can cause hormonal imbalances in the body, and this in turn predisposes a person to endometrial cancer [26, 42–44]. One of the risk factors for type 2 diabetes is obesity, which is also a major risk factor for endometrial cancer. Although the precise mechanisms and pathways are uncertain, it could be hypothesized that endometrial carcinogenesis is that exposure of the endometrium to excess estrogen unopposed by progesterone increases the mitogen activity of endometrial cells [45, 46]. In this meta-analysis the summary of RR of primary studies, whose results were adjusted based on BMI showed a less value compared to summary of RR of primary studies, whose results were adjusted based on weight control. In women with obesity the levels of estradiol and estrogen are higher than women with normal weight, [47, 48], and this could be one the possible reason for the increase risk of endometrial cancer because of obesity [48]. However, results of several studies showed that other factors, such as higher insulin levels and growth factors, may also increase the risk of endometrial cancer in women with obesity [49, 50]. Moreover, long-term insulin therapy may also be responsible for increased risk of endometrial cancer in women with diabetes (31).

In this study, the authors performed subgroup analysis based on type of primary studies, geographical area, and adjusted covariate. However, we could not perform subgroup analysis based on type of diabetes (type 1 and type 2) because the early studies did not specify or separate the types of diabetes. Diabetes is a chronic disease, whose diagnosis may not be accurate and specific, in which case it would lead to classification bias (non-differential misclassification). Therefore, the overall results obtained from primary studies should be interpreted with caution.

However most of included case-control and cohort studies in this meta-analysis controlled the variables of obesity and sedentariness but it is of utmost importance to consider the effect of confounding variables (sedentariness, hormonal disorders, and obesity) on determining the relationship between diabetes and risk of endometrial cancer in women. The major strength of this updated meta-analysis in compare to previous one is that more primary studies identified and included [8], therefore distinguished effects of diabetes on risk of developing endometrial cancer based on adjustments to BMI/weight presented with larger sample size (larger effect size).

### Limitations

Included primary studies did not mention the duration of diabetes and type of treatment (oral anti hyperglycemic agents and/or insulin). Furthermore, identifying women with diabetes in the primary studies was almost based on their self-reports. Since the primary studies did not consider type of diabetes therefore it was not possible to estimate the possible risk separately in in type 1 and type 2 diabetes.

## Conclusions

Diabetes seems to increases the risk of endometrial cancer in women, and this finding can be useful in developing endometrial cancer prevention plans for women having diabetes.

## Data Availability

Input data for the analyses are available from the corresponding author on request.

## Abbreviations

BMI: Body weight index
CI: Confidence interval
CINAHL: Cumulative index to nursing and allied health literature
EMBASE: Excerpta medica dataBASE
HR: Hazard ratio
IDDM: Insulin-dependent diabetes mellitus
MOOSE: Meta-Analyses of Observational Studies in Epidemiology
NIDDM: Non-insulin-dependent diabetes mellitus
OR: Odds ratio
RR: Risk ratio or relative risk
STROBE: Strengthening the Reporting of Observationally Studies in Epidemiology
